# Abnormal Resting-State Connectivity at Functional MRI in Women with Premenstrual Syndrome

**DOI:** 10.1371/journal.pone.0136029

**Published:** 2015-09-01

**Authors:** Qing Liu, Rui Li, Renlai Zhou, Juan Li, Quan Gu

**Affiliations:** 1 Beijing Key Laboratory of Applied Experimental Psychology, School of Psychology, Beijing Normal University, Beijing, P. R. China; 2 Department of Psychology, School of Social and Behavioral Science, Nanjing University, Nanjing, P. R. China; 3 Research Center of Emotion Regulation, Beijing Normal University, Beijing, P. R. China; 4 State Key Laboratory of Cognitive Neuroscience and Learning & IDG/McGovern Institute for Brain Research, Beijing Normal University, Beijing, P. R. China; 5 Center on Aging Psychology, Key Laboratory of Mental Health, Institute of Psychology, Chinese Academy of Sciences, Beijing, P. R. China; Southwest University, CHINA

## Abstract

**Objectives:**

Premenstrual syndrome (PMS) refers to a series of cycling and relapsing physical, emotion and behavior syndromes that occur in the luteal phase and resolve soon after the onset of menses. Although PMS is widely recognized, its neural mechanism is still unclear.

**Design:**

To address this question, we measured brain activity for women with PMS and women without PMS (control group) using resting-state functional magnetic resonance imaging (rs-fMRI). In addition, the participants should complete the emotion scales (Beck Anxiety Inventory, BAI; Beck Depression Inventory, BDI, before the scanning) as well as the stress perception scale (Visual analog scale for stress, VAS, before and after the scanning).

**Results:**

The results showed that compared with the control group, the PMS group had decreased connectivity in the middle frontal gyrus (MFG) and theparahippocampalgyrus (PHG), as well as increased connectivity in the left medial/superior temporal gyri (MTG/STG) and precentralgyrus within the default mode network (DMN); in addition, the PMS group had higher anxiety and depression scale scores, together with lower stress perception scores. Finally, there were significantly positive correlations between the stress perception scores and functional connectivity in the MFG and cuneus. The BDI scores in the PMS group were correlated negatively with the functional connectivity in the MFG and precuneus and correlated positively with the functional connectivity in the MTG.

**Conclusion:**

These findings suggest that compared with normal women, women with PMS displayed abnormal stress sensitivity, which was reflected in the decreased and increased functional connectivity within the DMN, blunted stress perception and higher depression.

## Introduction

Premenstrual syndrome (PMS) refers to a series of cycling and relapsing physical, emotion and behavior syndromes that occur in the luteal phase and resolve soon after the onset of menses [1]. About 5–8% of women suffer from severe premenstrual syndrome, most of these women also meet criteria for premenstrual dysphoric disorder (PMDD). PMS results from ovulation and appears to be caused by the progesterone produced following ovulation in women who have enhanced progesterone sensitivity [2]. While it is known that ovarian hormones have effects on the brain [3],few studies have examined to what extent ovarian hormones affect neural activity especially for women whose hormone fluctuations are abnormal (like PMS).

The women who suffer from PMS are often affected by negative emotions like anxiety, depression and irritability [4]. It has been indicated that most of these premenstrual complaints reported by women with PMDD are related to heightened stress-sensitivity [5]. Recent study further proved that the hypo-reactivity to stress predicted greater premenstrual symptom severity and the pattern of stress reactivity that distinguished menstrual cycle related mood disorders (MEMD) from non-MEMD was predictive of clinical symptomatology [6]. This may suggest that the PMS was a stress-related disorder and the underlying neural mechanism of PMS may be related to basic stress reactivity of the brain.

The stress-related disorders like the post-traumatic stress disorder (PTSD) showed symptoms on the neural activity level were mainly performing on the abnormal brain connectivity of resting state [7]. For example, Bluhm and his colleagues found that in the spontaneous brain activity of the default mode network (DMN), there were changes in the functional connectivity of the precuneus cortex (PCC) for PTSD patients in a resting state [8]. Lanius et al. observed that the anterior cingulate cortex (ACC)/PCC/prigenual ACC were functionally connected to the right amygdala for PTSD patients in a resting state, and the strength of this connectivity could predict the severity of the PTSD patients in the future [9]. The study by Sripada et al. further proved that, compared with the control group, the PTSD patients displayed decreased connectivity in the ACC/ventral medial prefrontal cortex (vmPFC) and hippocampus within the DMN [10]. Since the PMS was stress-related disorder, it would display abnormal connectivity within the DMN in the resting state just like the PTSD.

On the other hand, the PMS showed syndromes in the luteal phase and resolve soon after the onset of menses. Therefore, the menstrual cycle may influence the brain activity of PMS. Previous study have shown that menstrual cycle had effects on task-related brain activity for women with PMDD [11,12]. Gingnell and his colleagues found that compared to healthy controls, women with PMDD had enhanced bilateral amygdale reactivity when they were exposed to emotional faces in the follicular phase, but there were no difference between groups in the luteal phase [11]. While the study of Bannbers et al. showed that women with PMDD displayed decreased activity during both menstrual cycle phases (luteal and follicular) compared to healthy controls in several task-related parietal areas [12]. But few studies have investigated the extent to which the menstrual cycle can influence the behavior of functional networks at rest for women with PMS or PMDD. A recent study by Hjelmervik et al investigated the sex differences and menstrual cycle (menstrual, foliicular, and luteal phases) effects in resting state cognitive control network with fMRI and they found that the menstrual cycle effects on resting states were non-existent. They considered that the resting states were resting traits [13]. Therefore, in our study, we investigated whether and to what extent resting state functional connectivity is altered in women with PMS regardless of their menstrual cycle.

Above all, the purpose of the present study was to investigate the difference in DMN activity for women with PMS and healthy controls and to determine if the differences were related to the women’ anxiety, depression and stress perception. Our hypothesis was that compared with the healthy controls, the women wih PMS would present an abnormal pattern within the DMN which related to their blunted stress perception and higher anxiety and depression.

## Methods

### Ethics

All participants provided written informed consent to participate in this experiment. The experimental procedures were approved by the Institutional Review Board of the State Key Laboratory of Cognitive Neurosciences and Learning of Beijing Normal University. The study was performed in accordance with the Declaration of Helsinki.

### Participants

The participants were recruited via flyers in the university and campus network. Before the experiment, a gynecological examination and B-ultrasonic wave were used to eliminate organic diseases to confirm that the participants were healthy and had no medical and surgical diseases. At the same time, we adopted the premenstrual syndrome scales [14] and self-compiled basic information on the women’s MCs to screen and group the 86 females (18~30 years old). Exclusion criteria were as follows: being currently pregnant or lactating, taking oral contraceptives or being under medical treatment, abnormal personality, obviously psychological abnormal syndrome and no regular MCs. Finally, thirty-two women (22.88±1.74 years) completed the session of resting state fMRI. There were 16 women in the PMS group and 16 women in the control group. The women were tested once in two different cycle phases, i.e. the follicular phase (1–3 days after menstruation), and the luteal phase (1–3 days before menstruation). The demographic information of the participants is shown in Table 1. All participants had normal or corrected-to-normal vision. They were all right-handed, as determined by Chapman and Chapman’s scale [15].

**Table 1:** Demographic information for females in the control and PMS groups.

	PMS group	Control group
*N*	16	16
Age (years)	23±2	22±2
Menophania (years)	13±1	14±1
The phase of MC under testing	8 in LP, 8 in FP	8 in LP, 8 in FP
Length of MC (days)	29±2	28±3

PMS = premenstrual syndrome; MC = menstrual cycle; LP = luteal phase (1~3 days before menstruation); FP = follicular phase (1~3 days after menstruation). There were no significant differences in age, menophania, length of MC or phase of MC under testing for the females in the control and PMS groups.

### Materials

#### Self-Report Measures

Beck anxiety inventory and Beck depression inventory (BAI and BDI): The BAI consists of 21 items that assess the degree of anxiety. It utilizes a four-level scoring rubric in which 1 indicates no discomfort. The BDI also consists of 21 items, each of which presents a category. The description of each category divides it into four levels, and scores range from 0 to 3 for different levels. We used standardized scores for further analyses of the BAI; we calculated the total raw scores for the 21 items and then used the equation Y = int (1.19X) to transform them into a standardized score. We used the total raw scores of the BDI’s 21 items for further analysis. Both the validity and reliability of the Chinese version of the BAI and BDI are well established [16].

Visual analogue scale for stressful situations (VAS): A VAS is a 100-mm bipolar line that measures a characteristic across a continuum [17]. One end of the line is zero, indicating no stress, and the other end of the line is 10 cm, indicating unbearable stress. The middle portion of the line indicates varying degrees of stress. The participants marked a spot on the line resembling their subjective appraisal of stress perception. Scores were determined by measuring from the left end to the mark using a ruler.

### Procedure

The participants in the PMS and control groups were randomly arranged in the non-menstruation phase (luteal or follicular phases) to receive rs-fMRI. To confirm the relatively stable and low level of endogenous cortisol, all of the scanning tests were conducted between 14:00 and 17:00 pm. Based on the females’ physical characteristics and hormone level, the test date was set for the pre-menstrual phase (luteal phase), which was 1 to 3 days before menstruation, and the post-menstrual phase (follicular phase), which was 1 to 3 days after menstruation. For menstrual cycle stage verification, we obtained prospective self-reports about when their menstruation started and combined this information with the primary gynecological examination and B-ultrasonic wave results to arrange the test times. All the women completed the VAS (pre-VAS), BAI and BDI before the scanning. Then, they received rs-fMRI scanning for 8 minutes with their eyes closed. After the scanning, they filled in the VAS (post-VAS) again.

### Data Recording

#### Data acquisition

A 3-Tesla Siemens Trio scanner (Erlangen, Germany) at the Imaging Center for Brain Research of Beijing Normal University was used for image acquisition. Foam padding and headphones were used to limit head motion and reduce scanner noise. Functional images were collected using an echo-planar imaging (EPI) sequence with the following parameters: time repetition (TR), 2000 ms; time echo (TE), 30 ms; flip angle, 90°; field of view (FOV), 200 × 200 mm², 33 axial slices; thickness, 3.5 mm; gap, 0.1 mm; in-plane resolution (matrix), 64 × 64; and voxel size, 3.125×3.125×3.5 mm³. For each participant, 240 EPI functional volumes were collected. High-resolution structural images were collected using a 3D magnetization-prepared rapid gradient echo (MPRAGE) T1-weighted sequence with the following parameters: TR, 2350 ms; TE, 3.39 ms; flip angle, 7°; 144 slices; resolution, 256 × 256; and voxel size, 1×1×1.3 mm³. During the scan, participants were instructed to lie quietly, keep their eyes closed, and not to think of anything in particular.

#### Data preprocessing

Data pre-processing was performed using the Statistical Parametric Mapping program (SPM8, http://www.fil.ion.ucl.ac.uk/spm), Resting-State fMRI Data Analysis Toolkit (REST) V1.8 and Data Processing Assistant for Resting State fMRI (DPARSF) V2.0 Basic Edition (http://www.restfmri.net). The preprocessing included the following steps: intra-volume slice timing correction using the Sinc interpolation, inter-volume geometrical displacement correction using a six-parameter (rigid body) spatial transformation, between-subject spatial normalization into the Montreal Neurological Institute (MNI) anatomical space with 3×3×3 mm^3^ resampling, and spatial smoothing using a 4-mm full-width-half-maximum (FWHM) Gaussian kernel. Four participants were excluded because of excessive head motions under the criteria >2.0-mm in any direction or >2.0° in any angular motion during the scan.

### Data Analysis

#### Subjective reports of emotion and stress perception data analysis

SPSS16.0 software (SPSS Inc., Chicago, IL) was used to process and analyze data in the present study. We conducted a mixed-factor ANOVA of the group differences (PMS and control) in stress reactivity to the rs-fMRI scanning for which the test time (pre-scanning and post-scanning) was the within-subjects variable. All of the significant analyses used the two-way test (*p*<0.05), and the partial eta squared (*η* ^*2*^_*p*_) was the effect size. Independent-sample *t* tests were used for significant tests for the scores of BAI and BDI in different groups (PMS and control). For within-subject analysis, the Greenhouse-Giesser correction was used where appropriate. The data are all presented as the mean ± S.D.

#### fMRI data analysis

To define the DMN, first, we performed Group independent component analysis (ICA) on the preprocessed resting-state fMRI data using the Group ICA software in the fMRI Toolbox (GIFT, http://icatb.sourceforge.net). All data from two groups were grouped together into the GIFT. The Group ICA program included two rounds of principal component analysis (PCA) for reduction of fMRI data dimension, ICA separation, and back-reconstruction of the independent components (ICs) and the corresponding mean time course for each subject. Based on the minimum description length (MDL) algorithm, the optimal number that ICA used to spatially split the datasets into a final set of ICs was estimated to be 31. In the first round of PCA, the individual functional data of each subject was dimension-reduced to the optimal number. After concatenation across subjects, the functional data was again reduced to the optimal number through the second round of PCA. Then the data was decomposed by ICA using the Informax algorithm (*ref*:*Lee T-W*, *Girolami M*, *Sejnowski TJ (1999)*: *Independent component analysis using an extended infomax algorithm for mixed subgaussian and supergaussian sources*. *Neural Computation 11*:*417–441*). The component that covers the main regions of the DMN previously reported [1] was selected for statistical analysis. To improve the normality of intensity values in each DMN component map, Z-score scaling was applied to individual-subject spatial maps, normalizing them to zero mean and unit variance. Finally, to derive the within-group functional connectivity of the DMN pattern, a voxel-wise one sample t-test was performed with these individual DMN components. To detect regions with between-group differences, a two sample t-test was performed within the DMN patterns of the two groups. Clusters were considered significant at a combined voxel-extent threshold of an uncorrected voxel level of p < 0.01 and a cluster-extent threshold of a corrected p <0.05 using AlphaSim program (individual voxel p < 0.01). One sample t-test was analyzed in a 61×73×61 Brain Mask. Voxles showing significant connectivity in the two groups were defined as a DMN template, which was used as a mask in the two sample t-test.

#### Connectivity-behavioral analysis

To evaluate the association between the functional connectivity in the DMN and individual performance (such as BAI, BDI and VAS scores), a voxel-wise correlation analysis with the DMN was performed between the z scores of functional connectivity and behavioral measures Pre-VAS, BDI, and BAI (the standardized scores) in the PMS group. These associations were significant at a combined voxel-extent threshold of an uncorrected voxel level of p < 0.01 and a cluster-extent threshold of a p <0.05 using the Monte Carlo simulation with AlphaSim correction program (individual voxel p < 0.01).

## Results

### The subjective differences on emotion and stress perception for control and PMS groups

**Fig 1:**
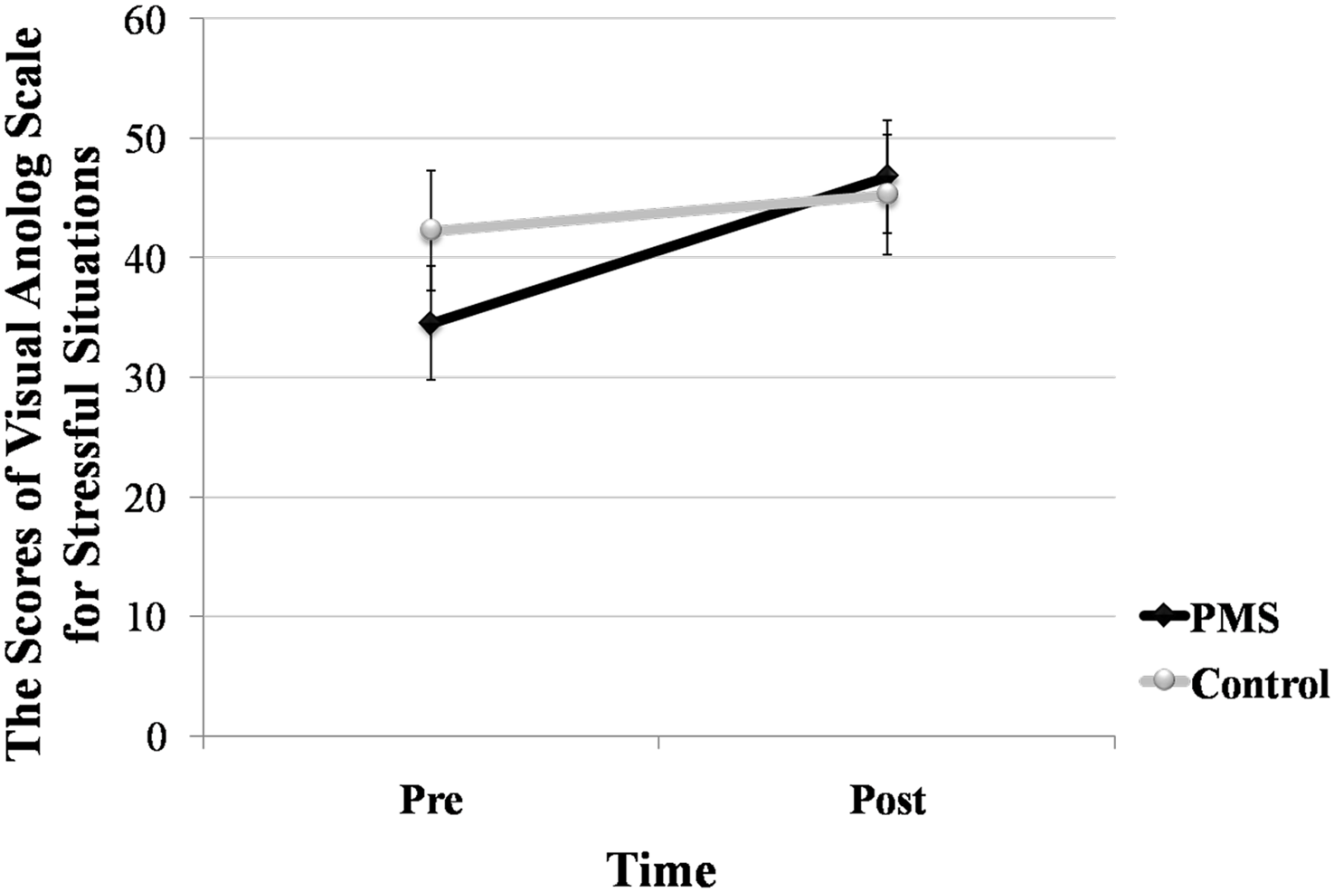
The scores of Pre-VAS (the scores on the visual analog scales for stressful situations before the scanning) and Post-VAS (the scores on the visual analog scales for stressful situations before the scanning) for females in the control (*N* = 16) and PMS (premenstrual syndrome, *N* = 16) groups.

A mixed-factor ANOVA was performed on the scores of the VAS. The within-subjects variable was the TIME (before and after the scanning), whereas the between-subject variable was GROUP (control, PMS). The results indicated that the main effect of time (*F*_(1,30)_ = 4.747, *p* = 0.037, *η2* = 0.137) was significant. Specifically, regardless of whether it was the control or PMS group, compared with pre-VAS, they achieved higher scores post-VAS, which are shown in Fig 1.

The independent sample *t* tests were conducted for the BAI and BDI scores for the control and PMS groups. The results revealed that compared with the control group, the PMS group had significantly higher BAI (*t*_(30)_ = 3.446, *p* = 0.002, *d* = 1.22) and BDI (*t*_(30)_ = 2.876, *p* = 0.007, *d* = 1.02) scores. The specific tendencies are shown in Table 2.

**Table 2:** The scores (*M*±*SD*) of BAI and BDI for females in the control (*N* = 16) and PMS (*N* = 16) groups.

	PMS group	Control group
BAI	33.25±4.89	8.81±4.83
BDI	28.00±3.63	4.19±4.25

BAI, beck anxiety inventory; BDI, beck depression inventory.

### Functional connectivity of the DMN for the control and PMS groups

#### Within-group functional connectivity of the DMN

**Fig 2:**
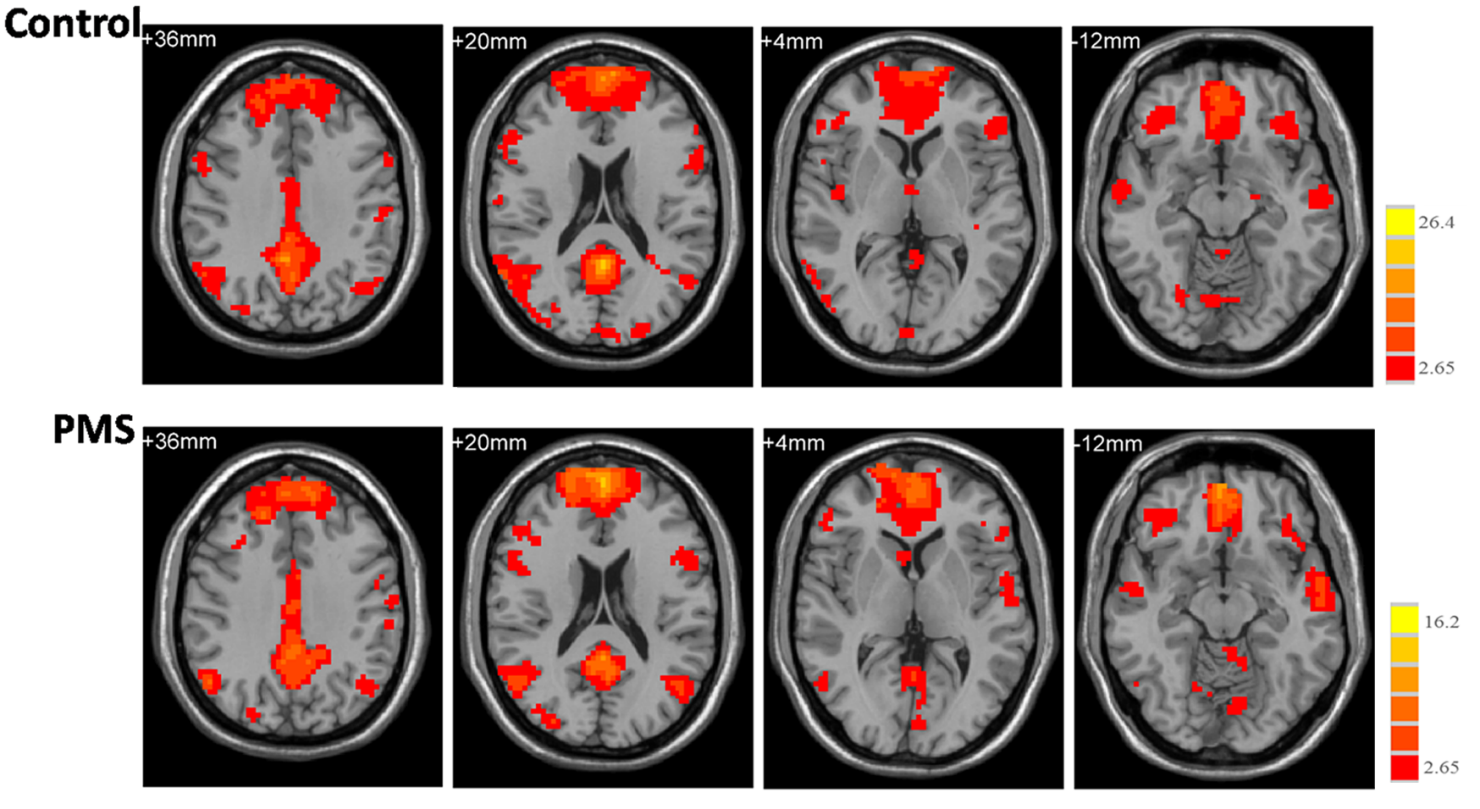
The functional connectivity map of the DMN (default mode network). Axial images show the network for the control (upper panel) and PMS (lower panel) groups. The statistical map was derived from a one-sample *t*-test of DMN components. The bar at the right shows the T-values. Images are in radiologic format with the left side of the image corresponding to the right side of the subject’s brain.

Fig 2 shows the functional connectivity map of the DMN extracted from the Group ICA. The regions displaying significant functional connectivity in both groups mainly cover the medial prefrontal cortex, middle frontal gyrus (MFG), precentralgyrus, precuneus/posteriorcingulate cortex, (para)hippocampal gyrus, superior/inferior parietal lobule, and the superior/middle/inferior temporal gyrus.

#### Between-group functional connectivity differences in the DMN

**Fig 3:**
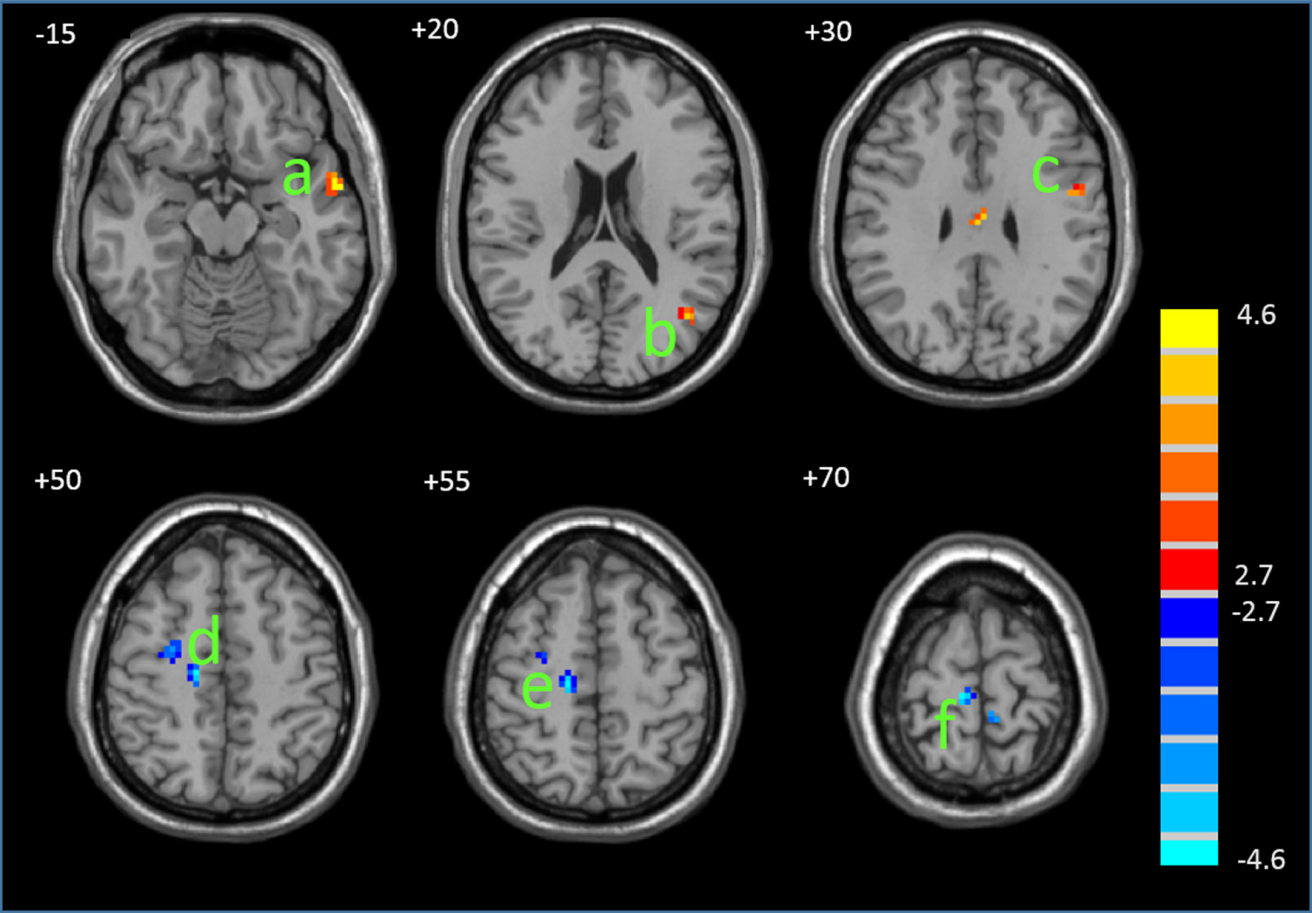
Differences in the functional connectivity between the PMS and control groups. The statistical map was derived from a two-sample *t*-test of DMN components. The red and blue color denote, respectively, the regions showing increased and decreased functional connectivity in the PMS compared with the controls. a, MTG/STG; b, PHG; c, MTG; d, precentralgyrus; e-g, MFG. The bar at the right shows T-values. Images are in radiologic format with the left side of the image corresponding to the right side of the subject’s brain.

Fig 3 displays the regions that displayed significant differences in the functional connectivity of the DMN between the PMS and controls. Compared with controls, the PMS group displayed significantly decreased functional connectivity in the MFG and posterior cingulate cortex and increased functional connectivity in the precentralgyrus and the left middle and superior temporal gyri (MTG and STG). Table 3 details the regions with between-group differences.

**Table 3:** Regions of DMN (default mode network) demonstrating altered functional connectivity in the PMS group.

	Brain regions	BA	Peak MNI location			Volume(voxels)	T value
			x	y	z		
Increased in PMS	MTG/STG	21/38	-57	0	-15	20	4.59
	MTG	39	-45	-69	18	13	4.02
	Precentralgyrus	6	-51	0	27	11	4.08
Decreased in PMS	PHG/posterior cingulate cortex	19/30	18	-51	6	28	4.48
	MFG	6	27	-6	51	20	3.89
	MFG	6	15	-21	54	18	4.53
	MFG	6	9	-27	72	40	4.56

### Connectivity-behavioral correlations

**Fig 4:**
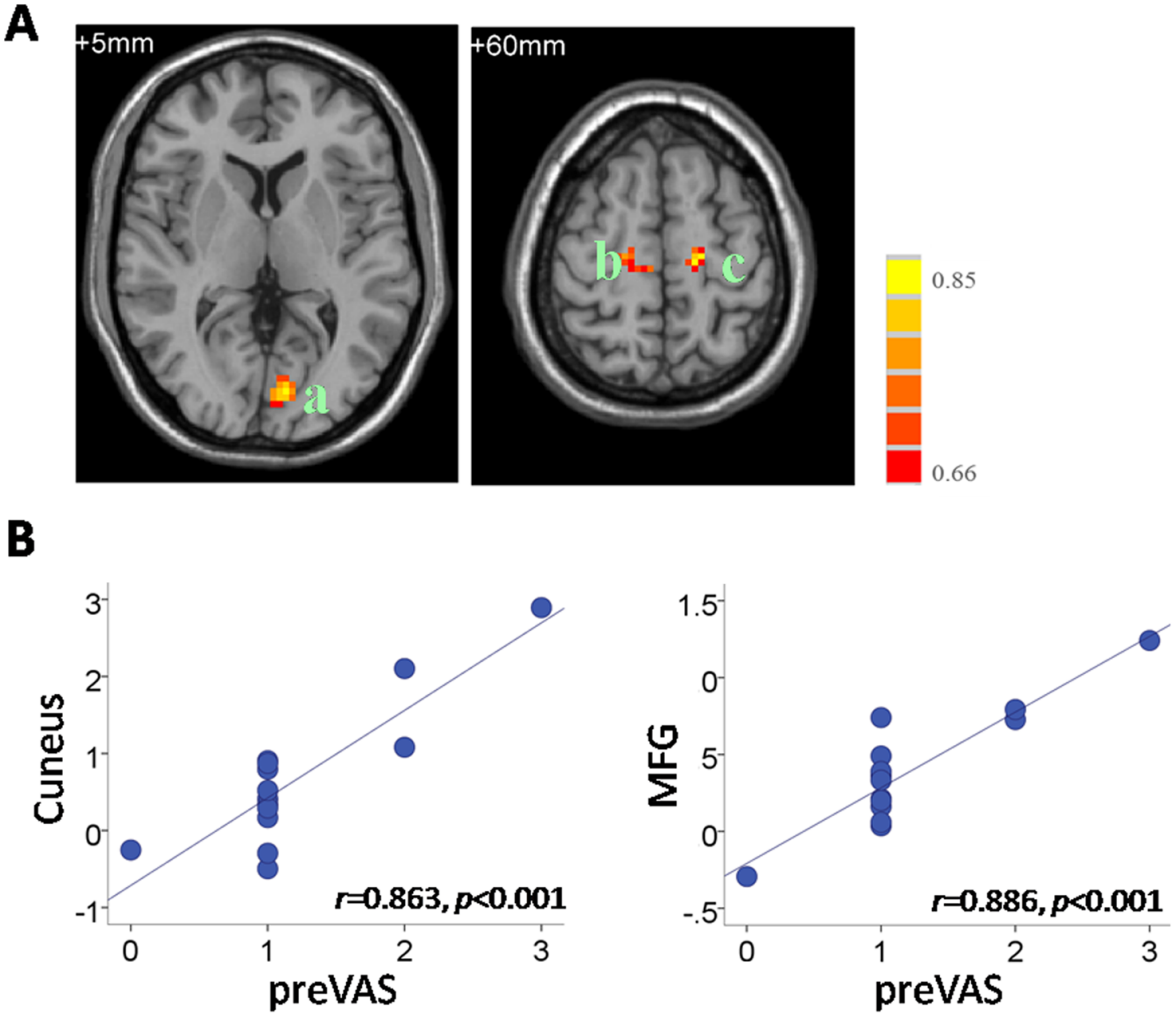
Correlation between the DMN connectivity and individual pre-VAS levels in the PMS group. (A) The regions showing significant correlations with pre-VAS. a, cuneus; b/c, MFG. Images are in radiologic format with the left side of the image corresponding to the right side of the subject’s brain. t. Bar at the right shows correlation (r) values. (B) Scatter plots of the correlations between pre-VAS and mean functional connectivity values in the cuneus (center: -3, 84, 3;6mm-radius sphere) and MFG (center: -18, -18, 60; 6mm-radiussphere). Each circular dot represents the data from one participant. The regression line indicates a positive relationship between the connectivity and the pre-VAS.

**Fig 5:**
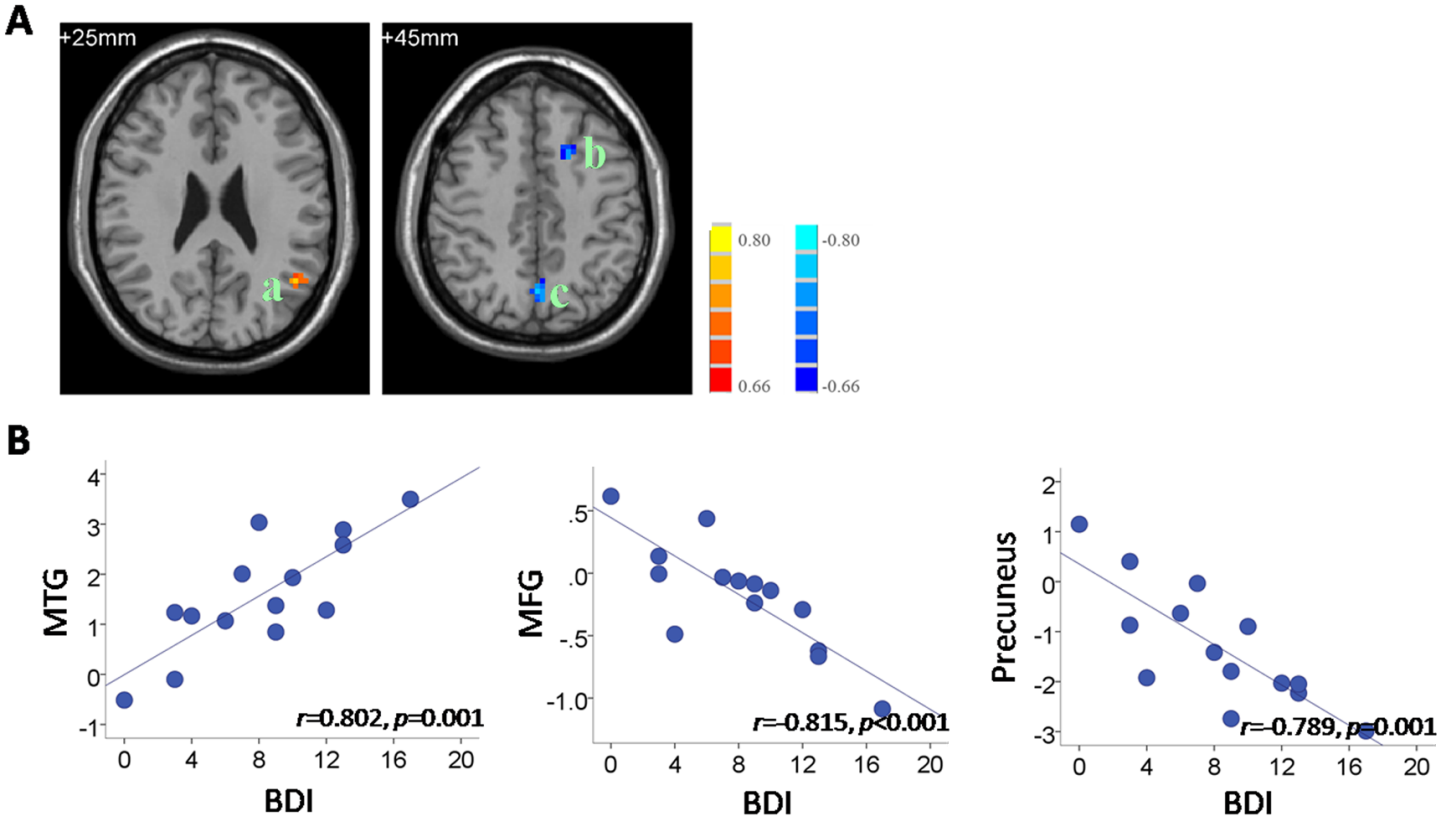
Correlation between DMN connectivity and individual BDI levels in the PMS group. (A) The regions showing significant correlations with BDI.a, MTG; b, MFG; c, precuneus. Images are in radiologic format with the left side of the image corresponding to the right side of the brain. Bar at the right shows correlation (r) values. (B) Scatter plots of the correlations between BDI and functional connectivity. Each circular dot represents the data from one participant. The regression line indicates a relationship between the BDI scores and the mean connectivity values in the MTG (center: -45, -60, 27; 6mm-radius sphere), MFG (MNI center: -18, 18, 48; 6mm-radius sphere), and the precuneus (MNI center: 6, -3, 42; 6mm-radius sphere).

Correlation analysis was applied to the functional connectivity in the DMN and individual behavioral data, including the pre-VAS, BAI and BDI in the PMS group. The results indicate significantly positive correlations between the pre-VAS level and functional connectivity in the MFG (*r* = 0.886, *p*<0.001) and cuneus (*r* = 0.863, *p*<0.001) (Fig 4). In addition, we found that the BDI scores in the PMS group correlated negatively with the functional connectivity in the MFG (*r* = -0.815, *p*<0.001) and precuneus (*r* = -0.789, *p* = 0.001) and correlated positively with the functional connectivity in the MTG (*r* = 0.802, *p* = 0.001) (Fig 5). No significant correlation between the BAI and functional connectivity was observed in the PMS group. Details on the regional clusters that displayed significant connectivity-behavioral correlations are listed in Table 4.

**Table 4:** Regions showing significant connectivity-behavioral correlations in the PMS group.

	Brain regions	BA	Peak MNI location			Volume(voxels)	R score
			x	y	z		
DMN-preVAS	Cuneus	17/18	-3	-84	3	53	0.86
	MFG	6/8	-18	-18	60	18	0.89
	MFG	6/8	18	-18	60	20	0.80
DMN-BDI	MTG	39/40	-45	-60	27	22	0.80
	MFG	8	-18	18	48	25	-0.82
	Precuneus	7	-3	-72	42	32	-0.79

## Discussion

The present study found that compared with healthy controls, the women with PMS displayed abnormal functional connectivity within the DMN that was independent of testing phase. Specifically, the PMS females displayed significantly decreased functional connectivity in the MFG and right PHG and increased functional connectivity in the precentralgyrus and the left MTG and STG. In addition, the anxiety and depression scores for women with PMS were significantly higher than those for healthy controls. Furthermore, compared with healthy controls, the women with PMS displayed increased scores for stress perception from pre-scanning to post-scanning. Finally, the results indicate significantly positive correlations between the pre-VAS level and functional connectivity in the MFG and cuneus. We also found that the BDI scores in the PMS group correlated negatively with the functional connectivity in the MFG and precuneus and correlated positively with the functional connectivity in the MTG.

The abnormal activations within the DMN for PMS females that we observed were in accordance with the perspectives of Brosschot [18]. Brosschot focused on the chronic stress and unconscious perspective cognition, which, in terms of brain imaging, indicates that prolonged physiological activation or changes in functional connectivity are markers of chronic stress. These results were consistent with the study of Sripada et al. [10]. Sripada and his colleagues found that compared with a control group, the participants in a PTSD group displayed abnormal activations in brain regions related to emotion and emotional regulation, which may be related to the pathology of PTSD. In our study, the functional connectivity within the DMN was separated for PMS females displaying decreased connectivity in the MFG and right PHG as well as increased connectivity in the precentralgyrus and the left MTG/STG. These results suggest that like the PTSD patients, the PMS females had relatively more sensitive brain activation to the threatening stimuli, and this activation still existed even when they were in a resting state not performing tasks. In addition, these results also confirm the viewpoint of Klatzkin et al. [6] related to stress. Klatzkin and his colleagues believed that the PMS females would display blunted reactivity to stress and may display a general reduction in performance due to stress such as participants with stress disorders. The present study further proved this viewpoint in the context of brain imaging and showed that PMS may be a mood disorder related to stress. In our study, there was abnormal functional connectivity within the DMN under the resting state, and this may reflect the PMS females’ supersensitive reactivity and sensitivity to stress.

Compared with the control group, the PMS group had higher anxiety and depression scores as well as lower stress perception scores. This confirmed again, from the perspective of subjective perception, that PMS is a stress-related mood disorder that results in general effects in patients similar to those in stress disorders such as PTSD. These results are in accordance with the study of Daniel et al. [7]. Daniel and his colleagues found that for the descriptive scores of subjective emotion, happiness and life events, the PTSD patients displayed significant differences in the BDI scores compared with the control group. However, our results were different from the study of Ossewaarde et al. [5], who found that there were differences in activation for females in different phases of the menstrual cycle. This was because our primary hypothesis was that as a stress-related disorder, PMS would produce changes in functional connectivity independent of the testing phase, and this reflects that PMS achieves prolonged changes in brain activation. We also obtained subjective results that were consistent with our hypothesis that compared with the control group, the PMS group displayed lower VAS baseline scores, which reflects their poor perception of stress, and then after the scanning, the PMS group had increased VAS scores, which suggested that they felt more stress; this may indicate that they had higher stress vulnerability.

With the help of the psycho-neural correlation analysis for the subjective and objective data in the present study, we found that before the scanning, the lower stress perception and higher depression were correlated with weaker functional connectivity in the MFG and PHG. This means that the abnormal emotion performance of PMS can be directly observed via brain imaging, specifically the decreased functional connectivity of rs-fMRI related to decreased emotion regulation ability. At the same time, the lower depression for the PMS group was related to stronger connectivity in the MTG, whereas compared with the control group, the PMS group displayed significantly stronger connectivity in the MTG and STG. These results are similar to the studies of Kirschbaum et al. [19] and Epperson et al. [20]. Their studies reported that females displayed stronger reactivity to psychological stressors when they were in the luteal phase, and this increased their negative mood, which was more obvious for the females with PMS. Their results emphasized how the natural fluctuations of hormones during the menstrual cycle were used to study the integration effects of gonadal hormones (progesterone and estrogen) on brain activity. Based on this research, our study further confirmed that the stress sensitivity and depressed mood for PMS females were related to their abnormal patterns of functional connectivity within the DMN. This finding may reveal the underlying mechanism of PMS and suggests that a follow-up study should investigate PMS from the perspective of stress-related disorders.

Overall, given the combination of the advantages of the resting state default mode network and the absence of related research for PMS females, we focused on the rs-fMRI of PMS and found abnormal pattern that may underlie the pathology for PMS. In addition, the abnormal pattern was independent of the menstrual cycle, which suggests that as one of the psychological disorders related to stress, there were functional disturbances in the DMN pattern for PMS that induced a permanent functional changes. These findings suggest that compared with healthy controls, women with PMS displayed abnormal stress sensitivity, which was reflected in the decreased and increased functional connectivity within the DMN, blunted stress perception and higher depression. These findings may contribute to the development of prognostic tools to distinguish between those who will and those who will not develop PMS from their resting data and emotional performances.
